# The Grahamite Theory

**Published:** 1876-07

**Authors:** 


Sylvester Graham was a genius in his way, and an enthusiastic one. His lectures were largely attended and have had hundreds of thousands of readers, under the title of "The Science of Human Life." That his teachings have been productive of good results, can not be doubted. He said that the miller who sifted or bolted his wheat meal, eating the white part and giving the darker portion to the quadrupeds, cheated himself. Multitudes believed this absolute truth, and began eating flours more or less fine, made of whole, unsifted grains. They got very valuable food by this means, but how they suffered! With the gray food-substance they took into the delicate stomach a fearful burden of indigestible trash, in the form of the outer hulls of the wheat, placed there by nature as a substantial wall of silex to protect the excellent food within. The human stomach could no more emulsify these harsh structures, than it could dissolve a mass of splintered glass. The camel, with his supplementary stomach, enjoys and demands them; the ruminating cattle are supplied with organs adequate for their digestion; the ass, with his leathery mucus membranes, disposes of them as he does of other nettles; the Indian, accustomed from infancy to the use of harsh, unprepared food, suffers no inconvenience from the use of grain and seeds and bugs and other insoluble substances. But as the human race rises in the scale of civilization and refinement, and evolutes beyond the crude methods of the lower orders, the digestive functions partake of the general elevation, and suffer from the intrusion of straw-like substances. With the general refinement there comes the necessity for refinement in our foods. Some things which the ox, the ape, the Indian, or the mere muscle-worker eat in comfort and without disadvantage, bring great misery, and fail to support the higher life of the brain-worker. The poet, the author, the advocate, the banker, the merchant, the orator, the artist, the man of mind, whose brain encompasses a world of thought each day, can no more live on locusts and wild honey, like the nomad of the desert, or on the husks with which the lower orders regale themselves, than he could thrive on chips and stones. His fine organism must be fed, and amply fed. The brain and its continuations, the nerves, demand food containing elements which are identical with brain substance, and this, if soluble, the stomach receives with gratitude, and all the organs of digestion appropriate with avidity. No truth is more clearly established among those who think closely, than this, viz.: that substances which the teeth and the stomach fail to reduce to a pulp, fail to emulsify; in other words, will not fail to do injury if taken into a stomach at all delicate or sensitive.

As a rule, there are no worse dyspeptics than the earnest disciples of Dr. Graham. It is natural that this should be so. The means employed will not fail once in fifty times. If we were anxious to drive a sedentary brain-worker into confirmed dyspepsia, indigestion, and constipation, we should prescribe saw-dust and aloes ; or, if we desired to accomplish the work very quickly and very effectually, we would feed him on wheat-bran, which would take the place of both. Saw-dust would induce fermentation in the stomach just as wheat-hulls do ; and wheat hulls would physic the bowels just as the drug does. It makes little difference whether your physic operates as a chemical or a mechanical cathartic. All accomplish the one result—that of compelling a copious secretion of fluid to protect the membranes against the irritant. The discharge of this fluid with the food-waste, is called purging, when considerable in quantity. This purgation will go on from week to week, and from one year's end to another, and still the physicked sufferer will live on. But he will be thin and weak, because there is a drain set up, which weakens and enfeebles. Men and women who are thus purged daily with bran or pills, become mere skeletons. If a vein were tapped every few hours and an ounce or two of blood withdrawn, the effect would be about the same.

Constipation will continue as long as food does not correspond to the employment and habits of life. The cure for it is not to be found in cathartics, whether they be wheat-bran, or rye-bran, or gamboge, or scamony, or aloes, or croton-oil. These do harm always. Local applications like the suppository devised by Nelaton of France, are safe and useful, and if supplemented by soluble nerve-food, will usually cure in a short time. The best food is that which, while being easily converted into good blood, leaves no hurtful, rasping waste to drain the system of its vitality as it is forced downward, and outward. To the preparation of just such food as the constipated dyspeptic and all other sufferers require, the Health Food Company of New York is devoting itself with intelligence, enthusiasm, and success. The army of American dyspeptics should consult them and learn their methods.

